# Supplementary material for: The causal relationship between the human gut microbiota and pyogenic arthritis: a Mendelian randomization study
Source: Front Cell Infect Microbiol. 2024 Nov 26;14:1452480. doi: 10.3389/fcimb.2024.1452480 (PMC11629706; doi:10.3389/fcimb.2024.1452480)
Supplement: Supplementary file 1 [file DataSheet1.zip › mendelian test/ebi-a-GCST90027574.csv_forest.pdf]

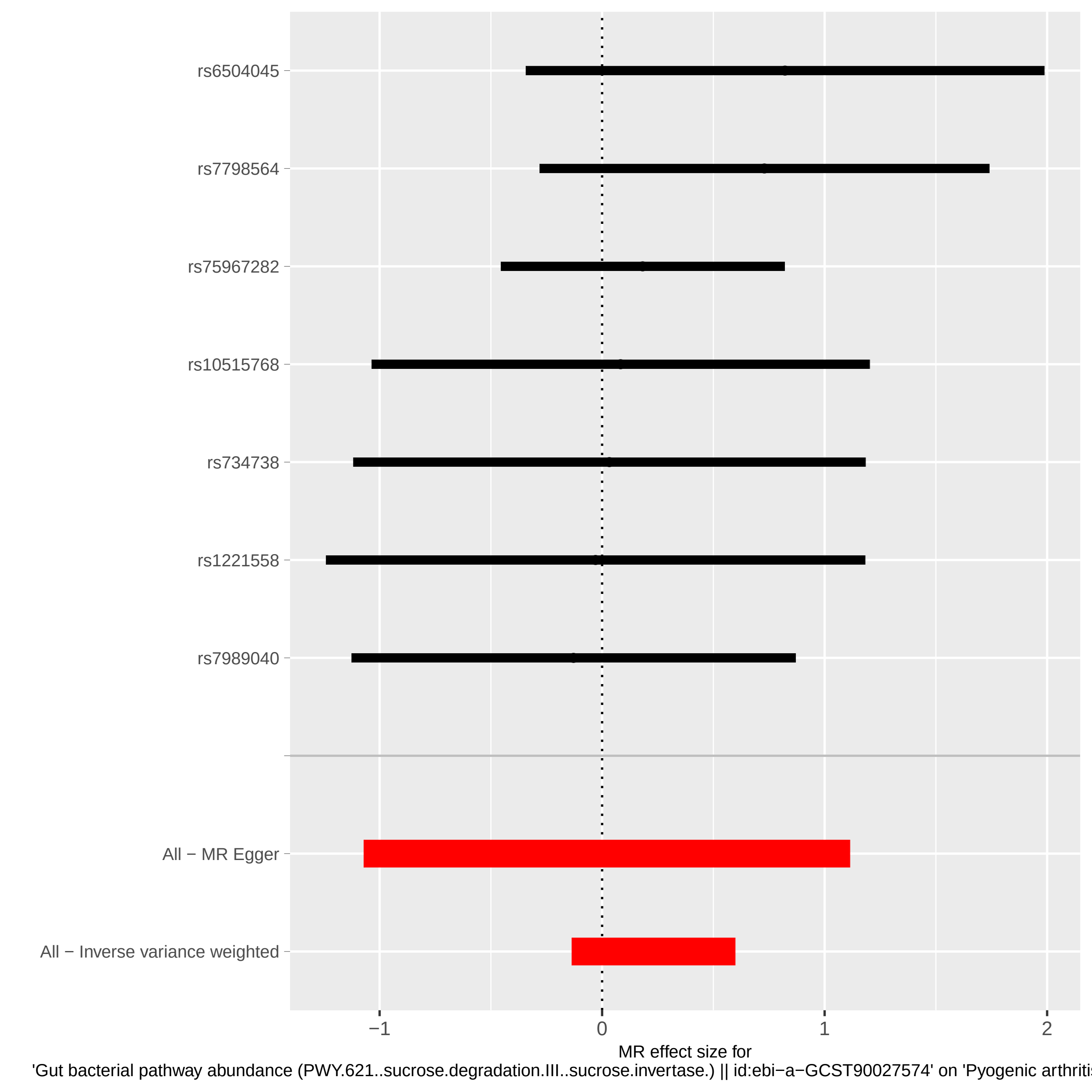
rs6504045
rs7798564
rs75967282
rs10515768
rs734738
rs1221558
rs7989040
All − MR Egger
All − Inverse variance weighted
−1
0
1
2
MR effect size for
'Gut bacterial pathway abundance (PWY.621..sucrose.degradation.III..sucrose.invertase.) || id:ebi−a−GCST90027574' on 'Pyogenic arthriti
